# Study protocol of a factorial trial ECHO: optimizing a group-based school intervention for children with emotional problems

**DOI:** 10.1186/s40359-021-00581-y

**Published:** 2021-06-21

**Authors:** Simon-Peter Neumer, Joshua Patras, Solveig Holen, Carina Lisøy, Anne Liv Askeland, Ida Mari Haug, Annette Jeneson, Tore Wentzel-Larsen, Frode Adolfsen, Lene-Mari Potulski Rasmussen, Jo Magne Ingul, Kristin Ytreland, Elisabeth Valmyr Bania, Anne Mari Sund, Kristin Martinsen

**Affiliations:** 1 The Center for Child and Adolescent Mental Health – Eastern and Southern Norway, Postboks 4623, 0405 Nydalen, Oslo, Norway; 2 The Regional Centre for Child and Youth Mental Health and Child Welfare – Northern Norway, RKBU Nord UiT Norges Arktiske Universitet, 9037 Tromsø, Norway; 3 The Regional Centre for Child and Youth Mental Health and Child Welfare – Central Norway, MTFS, Pb 8905, 7491 Trondheim, Norway; 4 St.Olavs Hospital, Trondheim University Hospital, Prinsesse Kristinas gate 3, 7030 Trondheim, Norway; 5 Department of Psychology, University of Oslo, Forskningsveien 3A, 0373 Oslo, Norway; 6 Norwegian Centre for Violence and Traumatic Stress Studies, 0484 Oslo, Norway

**Keywords:** Indicated prevention, Anxiety, Depression, Factorial design, MFS, Digital interventions, Parental involvement, Children, Effectiveness, Implementation

## Abstract

**Background:**

Youth mental health problems are a major public health concern. Anxiety and depression are among the most common psychological difficulties. The aim of this study is to evaluate an optimized version of a promising indicated group intervention for emotional problems. The program (EMOTION Coping Kids Managing Anxiety and Depression) targets school children 8–12 years with anxious and depressive symptoms and examines three factors. Factor 1 compares the standard EMOTION intervention delivered in 16 group-based sessions (Group), versus a partially-digital EMOTION intervention (DIGGI) delivered as eight group sessions and eight digital sessions. Both versions use virtual reality technology (VR) to improve behavioral experiments. Factor 2 compares parent participation in a 5-session parent group (high involvement) versus sharing information with parents via a brochure (low involvement). Factor 3 compares the use of a measurement and feedback system (MFS) designed to help group leaders tailor the intervention using feedback from children with no MFS.

**Methods:**

Using a cluster-randomized factorial design, 40 schools across Norway will be randomized to eight different experimental conditions based on three, two-level factors. To assess internalizing symptoms in children, children and their parents will be given self-report questionnaires pre-, post-, and one year after intervention. Parents also report on demographics, user satisfaction, personal symptoms and perception of family related factors. Teachers report on child symptoms and school functioning. Group leaders and the head of the municipal services report on implementation issues. The primary outcomes are changes in depressive and anxious symptoms. Some secondary outcomes are changes in self-esteem, quality of life, and user satisfaction. Questions regarding the consequences of the COVID-19 pandemic are included. Treatment fidelity is based on checklists from group leaders, and on user data from the participating children.

**Discussion:**

This study is a collaboration between three regional centers for child and adolescent mental health in Norway. It will provide knowledge about: (1) the effect of school-based preventive interventions on anxiety and depression in children; (2) the effect of feedback informed health systems, (3) the effect and cost of digital health interventions for children, and (4) the effect of parental involvement.

**Supplementary Information:**

The online version contains supplementary material available at 10.1186/s40359-021-00581-y.

## Background

Mental health problems in children are a major public health concern. Anxiety disorders are among the most common psychological difficulties, with studies indicating that 23.9% of children 6–8 years and 31.9% of adolescents have had an anxiety disorder at some point during their life [1]. Depression is also common, with studies indicating that up to 14.3% of adolescents have had a depressive episode [2]. Yet, anxiety and depression in children often go undetected in the specialist services in Norway [3]. Moreover, subthreshold anxiety and depression, which also considerably reduces daily functioning, may affect even larger numbers of children [4]. From 2015, the responsibility for treating mild to moderate depression and anxiety in Norway shifted from specialized mental health clinics to first line services [5]. There is, therefore, a great need for standardized assessments and interventions in first line services.

### Promising interventions for emotional problems

Developing integrated evidence-based interventions (EBI) that target multiple, but related, emotional problems (i.e., a transdiagnostic approach), has been an important task for the research group involved in the ECHO study. The study uses an optimized version of the EMOTION intervention [6]. The EMOTION intervention is based on cognitive behavioral therapy (CBT) with parental involvement. Our research group recently conducted a randomized controlled trial (RCT), the TIM study, in 36 schools (*N* = *873*) across seven municipalities in Norway [7]. Screening of 1686 children in this study identified 48% with high symptom levels of anxiety or depression. Outcome results were positive and indicated that the intervention can reduce symptoms of anxiety and depression and that the services would benefit from optimized screening and intervention methods [8–10]. International research supports these results [11], reporting medium to large effect sizes (0.38–0.79) for selective and indicated interventions. The same study suggested that further investigation is needed to optimize effects when interventions are transferred into routine practice.

ECHO explores three factors that might optimize effects: (1) combining the psychosocial intervention with a Measurement Feedback System (MFS), (2) combining traditional interventions and internet-based interventions, (3) including parents in the intervention.

#### MFS

In ECHO, group leaders can tailor the intervention for each child through a MFS. New results concerning the registration of personal aims in MFS for adolescents [12] indicate that MFS can support the service provider in enhancing the co-operation with youth and help them stay focused on the aims of the intervention. Results from a Cochrane report [13] on the effect of client feedback identified different MFS instruments that can be used in child health care. These instruments suffer, however, from use of measures that are not well-validated, have a rigid structure, are not adaptable, or are too costly in use for large-scale distribution [14].

#### Internet-based interventions

One of the areas that was highlighted by the group leaders in the TIM study was the overall length of the intervention. Meta-analyses indicate that internet-based interventions that reduce intervention costs have the potential to provide small to moderate effect-sizes when targeting anxious and depressive symptoms within traditional interventions [15]. To explore this further, the ECHO study evaluates the standard version of EMOTION with a revised, digital version (DIGGI) that conducts half of the sessions in an online format.

#### Parental involvement

Involving parents in CBT interventions targeting depression and anxiety in children can have a positive effect on treatment outcomes [16]. Yet, parental involvement has also yielded mixed results [17]. While it may be desirable to include parents in separate parent groups, resources to be able to run these groups are scarce in first line services. It can also be difficult for some parents to attend parent meetings.

The aims of examining these factors are to enable the group leader to tailor the intervention through feedback for the specific child, to facilitate implementation in the services, provide easier access for the children through digital sessions, and make behavioral experiments more accessible and realistic for the children through use of VR technology. Including parents may have the potential to support the intervention directed to the child. Integrating self-directed internet-based sessions along with traditional group meetings and the distribution of self-help materials to parents may reduce costs.

The elements mentioned above have not previously been evaluated together while optimizing an intervention. They constitute three factors and are related components that, properly combined, should form the strongest intervention outcomes. It is therefore useful that our multifactorial design allows for the testing of interactions as well as main effects (factors), in a design with equal distribution of all factors within each main effect [18]. Promising advances are also offered through use of new technology such as internet-based sessions and virtual reality (VR) technology. Behavioural experiments constitute an essential approach in CBT. VR technology that provides realistic situations can facilitate the child’s need for gradual exposure to situations that might otherwise result in avoidance or sadness. Through these VR situations, the group leaders establish stimulus control and similar situations can be rehearsed several times [19]. While not its own factor, the use of VR was included in both versions of the EMOTION intervention (Factor 2) because of its strong potential to improve outcomes.

## Method/design

The present study is a cluster randomized factorial design, with three factors, each with two levels, giving eight different experimental conditions randomized to schools across three regions in Norway: south-east, central, and north. Each of the 40 recruited schools will therefore be randomly assigned to one of the experimental conditions resulting in five schools per condition (see Table 1).

**Table 1:** Experimental conditions of ECHO project

	Factor		
Experimental condition number	1. Measurement feedback system (yes/no)	2. EMOTION coping kids (group/DIGGI*)	3. Parental involvement (high/low)
1	Yes	Group	High
2	Yes	Group	Low
3	Yes	DIGGI	High
4	Yes	DIGGI	Low
5	No	Group	High
6	No	Group	Low
7	No	DIGGI	High
8	No	DIGGI	Low

*The group version includes 16 sessions delivered in a group-format. DIGGI is a partially digital version with eight sessions delivered in group-format, and eight sessions delivered as internet-based sessions

The stages of the enrollment, intervention, and assessment can be seen in Table 2.

**Table 2:** SPIRIT table for evaluation of the ECHO study

	Study period						
	Enrolment^a^	Allocation^b^	After school allocation: Assessments and Intervention				Close-out
			Pre-I (T1)	Intervention	Post-I (T2)	Post + 1 yr (T3)	
Timepoint	Coh1: Q1 2020	Q1 2020	Coh1: Q1 2020		Coh1: Q2 2020	Coh1: Q2 2021	
	Coh2: Q3 2020	Q3 2020	Coh2: Q3 2020		Coh2: Q4 2020	Coh2: Q4 2021	
	Coh3: Q1 2021	Q1 2021	Coh3: Q1 2021		Coh3: Q2 2021	Coh3: Q2 2022	
	Coh4: Q3 2021	Q3 2021	Coh4: Q3 2021		Coh4: Q4 2021	Coh4: Q4 2022	
	Coh5: Q1 2022		Coh5: Q1 2022		Coh5: Q2 2022		Coh 5: Q2 2023
**Enrolment**							
Eligibility screen^c^			X				
Informed consent	X						
Allocation		X					
**8 interventions**				X			
**Assessments**							
Primary outcomes (MASC & SMFQ)			X^c^		X	X	X
Secondary outcomes except			X		X	X	X
User satisfaction, PIE Stigma evaluation sheet Implementation components					X		X
KIDSCREEN-27, RCADS, attitudes towards EBPs, TWQ, ICS, ILS			X				
Sustainability							X

^a^Enrollment occurs at the beginning of the semester prior to delivery of the intervention. Each cohort represents a group of children recruited during the semester

^b^Allocation (randomization) is conducted at the school-level, therefore allocation reflects when new schools joined the study

^c^Study eligibility for individual children is based on their scores on the MASC and MFQ (primary outcome measures)

*Coh* cohort, *Q* quartile

### Recruitment and eligibility criteria

School-aged children between eight and twelve years old from third to sixth grade are eligible for participation. The planned recruitment period of children and families lasts for a maximum of two and a half years, starting in spring 2020 until spring 2022. The recruitment of schools starts autumn 2019.

School eligibility will be determined by region of the country and municipality. School-size is a recruitment criterion, as the participating schools need to have at least one full class in each grade to increase the probability of recruiting enough children to run an EMOTION group. The study will be presented to key personnel (principals at schools, heads of different primary care health agencies) at each site. The person responsible for each site or health agency will sign a statement of intention to participate in the study, while interested schools will also sign an agreement to participate in the study and are asked to nominate a contact person. The intervention will be offered each semester (autumn/spring term) of each school year. The plan is to include an average of five children from each grade in each school every semester, but group size may vary with between three and seven children per group. The children are screened prior to participation (T1) and assessed after the intervention (T2) (see Table 2). One year after the end of the intervention, the children will complete the same self-report measures to identify possible long-term effects (T3), an important goal for all preventive interventions. Responsibilities of school contacts include informing teachers and parents at the targeted grades about the study and facilitating the completion of the child surveys at the measurement times (T1-T3). Children are presented with age-appropriate oral and written information about the study. Parents are informed about the study during parental meetings and receive a link to detailed written information and electronic consent. Parents are invited to consent on the child`s behalf if they consider that their child has more sad or anxious feelings than peers and the child wishes to participate in a group. Both parents (or one parent with single parent care) must provide a consent before a child can participate. Presentations and videoclips are available to help recruit schools and children. The recruitment of participants is reported in accordance with the Consolidated Standards of Reporting Trials (CONSORT) guidelines for clustered randomized trials [20]. A project webpage (https://www.echo.r-bup.no) is developed to enhance the communication between all participants.

#### Inclusion criteria

After informed consent is provided by the parents, children self-report on the Mood and Feeling Questionnaire – Short form for depression (SMFQ) [21, 22] and Multidimensional Anxiety Scale for Children (MASC-C) [23]. The MASC-C is a 39 items self-report assessing anxiety in children and adolescents between 8 and 19 years. The SMFQ [21] is a 13-items measure assessing cognitive, affective, and behavioral-related symptoms of depression in children aged 8–18 years, with one added question about suicidality. Children who score at least one standard deviation above the expected mean score for depression (SMFQ; *M* = 3.8, *SD* 3.6, inclusion ≥ 7 points), anxiety (MASC-C; girls *M* = 46, *SD* 15, inclusion ≥ 61 points; boys *M* = 39, *SD* 15, inclusion ≥ 54), or both, are then invited to join the study. Mean score on the MASC-C is based on Olason, Sighvatsson [24] and Villabø, Gere [25]. Mean score on the SMFQ is based on Angold, Erkanli [26] and Rhew, Simpson [22].

#### Exclusion criteria

There are few exclusion criteria. Children who may not benefit from a group intervention (e.g., language problems, severe cognitive or developmental challenges) are considered individually and exclusion is approved by the local PI. The reasons for exclusion are documented according to CONSORT guidelines.

### Intervention care and supervision

#### Intervention

The eight possible combinations of the factors MFS (yes/no), EMOTION (Group/DIGGI) and parental involvement (high/low), are used to create a unique intervention focusing on selected intervention elements.

#### Measurement-feedback system (MFS)

In close cooperation with the University of Oslo, we have developed the MittEcho App (Norwegian, MyEcho translated) to address limitations of existing MFS, including the use of not well-validated measures and a monetized approach. The MittEcho App is freely accessible and compatible for most versions of IOS and android operating systems. MittEcho gives children the opportunity to assess their own progress by evaluating three idiographic goals established by the children, and by measuring their symptom levels derived from the Behavior and Feeling Schedule ([27]; 6 items). All MittEcho results are sent from the App to a secure, digital platform; The Service for Sensitive Data (TSD). The MittEcho publication portal is part of TSD and is a platform that group leaders access to gain access to graphical displays of the participants’ collected feedback. Group leaders apply for secure access to the MittEcho publication portal using their national, digital ID. The data storage and sharing services at TSD comply with the General Data Protection Regulation (GDPR).

#### Group (16 sessions) versus a partially digital (8 + 8 sessions) EMOTION version

The indicated transdiagnostic intervention EMOTION [28] has been revised for this study to an intensive course that runs over 16 sessions for 8 weeks. EMOTION is based on CBT. The first half of the intervention focuses on building skills, psychoeducation, emotion regulation, and behavioral activation. The second half focuses on practicing skills, maintaining activation, cognitive restructuring and behavioral experiments. In the traditional group version, all 16 sessions are conducted in a group-setting at school lead by two trained group leaders. In the partially digital version (DIGGI), eight selected sessions are conducted in a group setting, while eight are delivered as internet-based automated sessions in which children work on their own at home (approximately every other session internet-based). The digital sessions created for DIGGI are child-friendly and provide examples and possibilities for rehearsal of coping strategies through fun activities requiring little writing. For the purpose of this study, DIGGI sessions are published on a platform that enables the collection of user data.

Both versions of the revised EMOTION intervention use VR during group sessions to conduct behavioral experiments in addition to in-vivo behavioral experiments. VR has the potential to create realistic situations using 3-D videos with head-mounted display to support behavioral experiments. For this purpose, several short VR films have been created, showing challenging scenes in the daily life of a school child (e.g., classroom presentation with different levels of difficulty to accommodate the child’s need to handle difficult feelings and approach avoided situations). Each VR film generates questions to the child about feelings and thoughts in the situations.

#### High versus low parental involvement

In high parental involvement condition, parents will be invited to participate in a 5-session EMOTION parent group [29]. In three of the five parent sessions, the child participates together with the parents. The groups will be running parallel to the child sessions with similar and complementary topics. The parenting sessions focus on creating a supportive home environment for the children as well as transferring and practicing new skills together with the children in joint group meetings. In the low parental involvement condition parents are presented to a self-help brochure [30] providing age-appropriate information and advice about anxiety and depression.

#### Supervision

To support the group leaders in the delivery of the intervention conditions, they will attend regular supervision conducted by a trained cognitive behavioral supervisor. The supervisors, in turn, will receive support from the study coordinating central office (RBUP East and South). Group leaders are also asked to complete fidelity check lists for each session of the intervention.

### Procedure

The study is registered at clinical trials (NCT04263558) and is funded by a grant from the Kavli Trust Programme on Health Research (project number 31/18). Additional funding for the project is provided by The Center for Child and Adolescent Mental Health – Eastern and Southern Norway (RBUP East and South) and The Regional Centers for Child and Youth Mental Health and Child Welfare – Northern Norway and Central Norway (RKBU North and RKBU Central, respectively).

### Randomization

Participants are randomized at the school-level. The schools are randomized into one of the eight different experimental conditions. Each school agrees on participation for approximately five semesters, entailing five waves of data collection. The school randomization procedure is carried out in the statistical software R, using a function specifically written for the ECHO study by the project statistician in collaboration with key personnel. The actual randomization (running the R function) is witnessed by an objective third party. The function takes two important aspects into account: (1) a balanced distribution of schools to the eight factors, even if the number of schools will be uneven, e.g. in case of new schools, and (2) a balanced distribution of the varying workload caused by the different conditions. The latter is to assure that it is not possible to overload a service provider with assignment of multiple resource demanding intervention conditions.

The randomization procedure was performed in two steps. In the first step, the municipalities were allocated to the two most demanding conditions. These municipalities were randomly drawn proportional to the number of schools within the municipality to ensure that each school had approximately the same probability of being allocated to one of these conditions. After the municipalities were selected, one random school was selected within each municipality. In stage 2, the remaining schools were allocated to the remaining conditions. The function that was used first sorted schools by size, then divided them into size groups equal to the number of remaining conditions. If one of the remaining conditions was allocated to only five schools, one size group was subsequently selected to have this condition reduced. Within the size groups the schools were randomly allocated to the remaining conditions so that the correct number of schools are allocated to each condition.

For practical reasons, the EMOTION groups are limited to seven participants. When too many children are eligible based on the results of the screening procedure, seven of the eligible children are randomly invited to child-groups. The randomization is performed through an automated process in our data collection system through which the children also respond to the screening survey. The procedure ensures that it is random which of the eligible students are first offered to be included. It is important that also this selection is random, and not based on non-random factors such as who first completed the assessment, assessment scores, or a school contacts personal assessment of who (out of a list of eligible students) might benefit most from the intervention.

The parents of the children who are excluded through this randomization procedure, are informed why their child is not invited to a group.

### Blinding

Because of the nature of the current interventions, it is not possible for the participants to be unaware of the intervention condition they are assigned to after baseline. Furthermore, schools are randomized to one of the conditions for the entire project period. Therefore, most of the participants are aware of the treatment condition prior to the baseline assessment.

### Outcomes

Data are collected at three time points: T1 (pre intervention), T2 (post intervention) and T3 (1-year post intervention follow-up). Parents, teachers, group leaders, and service leaders receive unique links to electronic surveys via email or SMS. Children use unique, confidential identifiers to access the child-survey using computers at school. Because of the children’s ages, an adult is present while they complete the questionnaire in order to clarify potentially confusing questions. Both parents are asked to complete the parent survey separately.

All measures have good psychometric properties and have been adapted and used in Norway as well as in international studies. We will use symptom measures that are free of charge (e.g. Revised Children`s Anxiety and Depression Scale, RCADS, 31) together with established copyright protected instruments (e.g. MASC), aiming to develop measures and a MFS system with good psychometric properties that is free to use for all service providers.

#### Primary outcomes

The primary outcomes of interest for this study are changes in level of depression and anxiety from pre- to post-intervention and from pre-intervention to follow-up. The null hypotheses for the primary outcomes of this study are that there will be no differences in changes of depression or anxiety scores between the three factors (main effects). The primary outcome measures will be based on two informants: children and parents.

Depression is assessed using the SMFQ [21], a 13-item measure assessing cognitive, affective, and behavioral-related symptoms of depression in children 8–18 years. Anxiety is assessed using the MASC-C/P [23], a 39-items self-report measure for adolescents between 8 and 19 years. Parents will use the parent version of the same instruments.

#### Secondary outcomes

See Table 3.

**Table 3:** Table for secondary outcomes in the ECHO study

Instrument	Description
*Beck self-Concept Inventory for Youth* (BSCI-Y)	The BSCI-Y [36] is a 20-item 4-point Likert subscale from *Beck Youth Inventories—Second Edition* (BYI-II). The BSCI-Y subscale measures perceptions about self-competence, potency, and positive self-worth. Reported Cronbach`s alpha for BSCI-Y is .83-.92 [37]
*General Self-Efficacy Scale* (GSE-6)	The GSE-6 is a short-form of the GSE [38], a psychometric scale designed to assess optimistic self-beliefs to cope with a variety of difficult demands in life. The GSE-6 has shown satisfactory validity and reliability with reported Cronbach’s alpha between .79 and .88 [39]
KIDSCREEN-27	The KIDSCREEN-27 [40] is a 27-item scale used to assess generic health-related quality of life. The scale has five dimensions: Physical Well-Being, Psychological Well-Being, Autonomy & Parents, Peers & Social Support and School Environment. Internal consistency values (Cronbach's alpha) range between .79 (Physical Well-being) and .84 (Psychological Well-being) for the different dimensions for the self-report versions [41]
*Revised Child Anxiety and Depression Scale* (RCADS C/P)	The RCADS C/P [31] is a 47-item, children self-report questionnaire. Convergent and discriminant validity tests are favorable, and the measure has showed greater correspondence to specific diagnostic syndromes than traditional measures of anxiety and depression [42]
*Behavior and Feelings Survey* (BFS)	The BFS [27] is a brief, 12-item rating scale designed to facilitate efficient progress-monitoring during youth psychotherapy. Items are rated on a scale from 0 (not a problem) to 4 (a very big problem). Three scale scores can be derived: Internalizing Problems (sum of items 1–6), Externalizing Problems (sum of items 7–12), and Total Problems (sum of items 1–12). Item 1–6 will be used in the MittEcho App
*Stigma and Evaluation sheet*	The *Stigma and Evaluation sheet* [43] measures overall user-satisfaction and participants’ experiences of stigmatization. A Norwegian translation of this 10-item instrument with a 10-point scale is used
*Idiographic aims*	The *Idiographic aims* are based on a self-report assessment tool for adolescents [44] and have been adjusted for use with children in the ECHO study. The instrument enables the respondents to define their most important aims in the MittEcho App to be addressed during sessions
*COVID-19 questions*	The *COVID-19 questions* are 9 self-constructed questions for parents and children measuring the impact of COVID-19, see Additional files 2, 3
*Brief problem monitoring* –teacher (BPM-T)	The BPM-T is an 18-item version of the Child Behavior Checklist scale (CBCL). The CBCL is a component of the *Achenbach System of Empirically Based Assessment* (ASEBA, 45). The BPM-T provides a uniform problem scale assessing both behavioral and emotional problems in school with good reliability and construct validity [46]
*Teacher’s Report Form* (TRF)	Academic performance and school adaptation measures are based on an adapted version of two factors from TRF. The TRF is a component of the ASEBA [45] and is used to investigate problem areas as well as academic achievement and adaptive functioning at school
*Performance in the academic subjects*	The *Performance in the academic subjects* Norwegian, English, mathematics, and social studies will be rated by the teacher on a scale from 1 (far below mean) to 5 (far above mean) compared to children of the same age. Based on these ratings a mean score will be calculated representing academic performance. *School adaptation* is based on evaluations of four elements: how hard the child is working, behavior, learning and how happy the child seems to be compared to children of the same age. The scale ranges from 1 (far below mean) to 5 (far above mean) and a mean score will be calculated
*Attitudes towards evidence-based practice* (EBP)	Six questions regarding attitudes towards evidence-based interventions will be asked. These questions are previously used in a large Norwegian study, investigating important factors related to cooperation and quality in services for children and their families [47]
*Total workload Questionnaire*, (TWQ)	The TWQ [48] includes 15 questions regarding autonomy and workload that will be used to assess these important parts of the group leader’s work situation
*The Implementation Climate Scale* (ICS)	The ICS [49] will be used to assess the implementation context in the participating agencies. The original psychometric properties are good, and the instrument is currently being investigated in a Norwegian context [50]
*The Implementation Leadership Scale* (ILS)	The ILS [51] will be used to assess to what extent the leaders in the services support and promote implementation of evidence-based interventions. The psychometric properties have shown excellent internal consistency and convergent and divergent validity for the original version
*Post-intervention questionnaire* (PIE)	The PIE, a self-developed questionnaire (see Additional file 1) regarding experiences and satisfaction with running EMOTION groups. After the groups are finished, the group leaders will be asked to evaluate their experience running the groups, level of supervision, and how satisfied they are in general with the manual and conducting the intervention. Additionally, we will ask more specifically questions regarding the group leader’s experience with the different factors and use of VR technology
*Implementation components*	From the Measures of Implementation Components [52], we include 43 questions regarding “implementation climate” at post group and one year after finishing the last group to assess how the intervention is integrated into the service
*Sustainability*	One self-developed question regarding sustainability (“Are your service still running EMOTION groups?”) with a follow-up question on “why/why not” will be included one year after the groups are finished to both group leaders and service leaders
*Columbia Impairment Scale* (CIS)	The CIS parent version [53] is a 13-item scale assessing general impairment in various functional domains, including relations with family members at home, relations with peers, school functioning, and involvement in general interests and activities. The CIS has shown high internal consistency (a = .89) and test–retest reliability (ICC = 0.89) and strongly correlates with other outcomes, such as mental health service utilization [53, 54]
*Parenting to Reduce Child Anxiety and Depression Scale* (PaRCADS)	The PaRCADS [55] is a 79-item, 10-subscale instrument developed to assess parenting and 5 of the scales are used in the present study: Rules and consequences for child, Health habits, Managing emotions, Setting goals and dealing with problems, Dealing with negative emotions. Items are scored on a five-point scale (almost never -almost always), or, for hypothetical questions (very unlikely—very likely). The instrument has shown adequate psychometric properties in one study
The *General Functioning subscale of the Family Assessment Device* (FAD)	The FAD [56] is a 12-item, self-report designed to map families healthy and unhealthy functioning using the General family functioning subscale. Each item is rated on at 4-point scale from strongly agree to strongly disagree. Previous research has shown high validity and test–retest reliability for the scale [57]
*Parental Bonding Instrument* (PBI)	The PBI [58], is a 16-item scale, answered by parents containing three subscales: warmth, authoritarianism and protection [59]. Psychometric properties of the PBI have been reported in a Norwegian study by Rimehaug and colleagues [60], with alpha coefficients ranging between .76 and .86. Validity has been established in several studies, and the stability of the instrument has been shown to be good
*Hopkins Check List* (HSCL-10)	The ten-item HSCL-10 has shown good psychometric properties [61]. It has also demonstrated good sensitivity and specificity for detecting psychological symptoms. The HSCL-10 consists of 10 items on a four-point scale ranging from 1 = ‘not at all’ to 4 = ‘extremely’
*Cost data*	*Cost data* will be collected regarding direct costs related to material, training, trainer payment, supervision, salary to group leaders for attending training, supervision, conducting sessions, and costs with the technical MFS platform. Costs regarding child use of mental health service, time lost from work for parents will also be estimated

*Demographic information* is collected from the service provider, group leaders, children and parents.

#### Recruitment and participation

Recruitment and participation data will be reported for available data from baseline.

#### Participant retention

Contact with participants’ families is maintained by school personnel and automatically generated reminders to complete questionnaires are sent via email or SMS.

#### Data management

Data will be obtained via electronic surveys from parents, teachers and children, using web-based questionnaires at baseline (T1), immediately after the intervention (T2), and at 12-month follow-up (T3). Data from group leaders and service leaders will also be obtained through electronic surveys, but at different time points than the other informants. Data are collected and managed by an independent data collection team at the study coordinating central office (RBUP East and South). The systems meet requirements for data security and EU’s data protection regulation (GDPR). Data analysis and data cleaning will be performed by the study investigators. Data are stored on a secure server during the study and analysis of results. Project staff have access to the final trial dataset. Upon completion of the study, the data are anonymized and archived according to Norwegian law. Data collected with the Mitt Echo App will be obtained every week of the intervention from children in schools assigned to the MFS condition. User data (e.g. time and duration of use) from the children and group leaders in this condition will be accessible. Canvas (a learning and communication platform) will produce user data from children assigned to the digital condition (DIGGI) and be collected.

### Data analysis

The study is carried out in schools and therefore data analyses will be conducted in a multilevel modeling framework to account for non-independence of the participants at the school level.

#### Sample size

The power estimation was based on an assumption of independence in the data (see Table 4, line A). The power was then adjusted on the need for a multi-level approach, as cases will be clustered in schools (see Table 4, line B) [32, 33]. The desired significance level is *p* = 0.05 and power is 0.80. The following additional conditions were accounted for: (1) anticipated effect size (*d*) of the main factors: 0.25 (conservative estimate), (2) the intraclass correlation coefficient (ICC): 0.05 (estimate is based on the level of school affiliation in Norway) [34], (2) average size of the clusters = 20 (i.e.: number of individuals within each school in a 24-month intervention period). Ukoumunne and colleagues’ equations ([33]; page 31 and 32) and Altman’s recommendations are used [35].

The total of individuals should be at least N = 796 across 40 schools. With eight experimental conditions, 5 schools will be assigned to each condition. With 40 schools, 20 children per school will be needed (see Table 4, line B).

**Table 4:** Number of participants and clusters required in a multilevel study

	Assumed ICC	Design effect	*N* indiv	*n/*cluster	*N schools*
A	0.00 – Base model	1	400	20	20
B	0.05 – Two-level model	1.95	800	20	40

Design effect = 1 + (n_c_ − 1)

*ICC* intraclass correlation coefficient, *n* number of pupils

*ICC (n_c_ = average number of individuals in a cluster = 20)

### Planned statistical analysis

Several models will be run to test for the main treatment outcomes, implementation outcomes, and related research questions.

The analyses will involve changes in outcome variables. A combination of linear mixed models and latent growth curve analyses will be used to identify differences between treatment groups, as well as change rates in growth over time. Based on the experience from the previous study, the rate of missing data due to electronic data collection will be low. Otherwise, missing data will be accounted for using full information maximum likelihood in growth curve analyses and restricted maximum likelihood techniques in mixed effects models where applicable, well-established techniques that allow for the inclusion of all available data.

To investigate the psychometric properties of some instruments initial exploratory factor analyses will be performed to investigate which factors we are able to obtain from our sample. Further, confirmatory factor analysis will be used. Correlations between subscales will be computed using Pearson’s r. Internal consistency for the subscales will be measured with Cronbach’s alpha.

#### Data monitoring committee

There is no formal establishment of a data monitoring committee for this project; this is generally not considered a necessary function for a research project of this type.

#### Cost

The costs of the interventions are evaluated by calculating direct costs related to material, training, trainer payment, supervision, salary to group leaders for attending training, supervision, conducting sessions, and costs with the technical MFS platform. An estimate of per-child costs is included in the final report to funders, along with additional estimates of costs incurred by the trial research team.

## Discussion

The present study will promote much needed research and innovation for optimizing service provision in first line health services to aid the alarmingly high number of school children who suffer from clinical and subthreshold levels of anxiety and depression. The effect of different versions of an evidence-based intervention for these children will be assessed using a cluster randomized design involving 40 schools across Norway. The aim is to create a framework that allows more evidence-based psychosocial interventions to be provided at a lower cost to society.

### National collaboration

The study is an active collaboration project between three regional centers in Norway responsible for work with mental health problems among children and adolescents: RKBU-north, RKBU-central, and RBUP east and south.

### International collaboration

Professor Philip C. Kendall at Temple University in Philadelphia, PA, USA and Dr. Linda Collins have been involved in the project planning, design and are involved in advising, data analysis, and publication of results.

## Trial status

The trial began recruiting schools in autumn 2019 (Actual Study Start Date, February 13, 2020) and is continuing through spring of 2022. Data collection pre/post will finish in spring/autumn 2022 with the last follow up assessment autumn 2023.

Trial registration: Clinical Trials NCT04263558, first posted on February 11, 2020, last updated on January 25, 2021.

*Secondary registration*: Kavli Trust Programme on Health Research (31/18).

*Primary sponsor*: The Center for Child and Adolescent Mental Health – Eastern and Southern Norway (RBUP East and South), Gullhaugveien 1–3, 0484 Oslo, mail@r-bup.no and.

*Protocol version*: November 2020.

## Ethics and dissemination

### Changes to the protocol

Changes to the project are made in the Standard Operating Procedures. These changes are recorded and maintained by the central investigator from RBUP East and South. Changes which are not merely procedural but may impact the experience of the participants in the study are reported to the Regional Committees for Medical and Health Research Ethics for approval.

### Confidentiality

Study participants are provided study IDs. A study key with the participant’s name and ID are stored in a secure internal server at RBUP East and South, separate from the participants survey answers and user data. That is, researchers only access and analyze deidentified personal data. Reporting of outcomes will be done using aggregate data.

*Contact for scientific inquiries* should be addressed to the central scientific investigator, Simon-Peter Neumer, simon-peter.neumer@r-bup.no.

*Dissemination of results* will be through scientific publications, the project webpage, reports to the funder, and press releases to news media. Three Ph.D. students who are part of the project team will publish and publicly defend dissertations relating to the study. Planned scientific publications include primary outcomes, secondary outcomes, psychometrics, and implementation results. The project team has adopted the Vancouver Protocol for determination of authorship of scientific publications.

## Supplementary Information

**Additional file 1**. Post-intervention questionnaire (PIE).

**Additional file 2**. COVID-19 questions child.

**Additional file 3**. COVID-19 questions parents.

## Data Availability

Datasets will be available from the corresponding author on reasonable request.

## Abbreviations

ASEBA: Achenbach System of Empirically Based Assessment
BFS: Behavior and Feelings Survey
BPM-P/T: Brief Problem Monitoring-parent/teacher version
BSCIY: Beck Self-Concept Inventory for Youth
CBCL: Child Behavior Checklist
CBT: Cognitive Behavioral Therapy
CONSORT: Consolidated Standards of Reporting Trials
COVID-19: Coronavirus disease 2019
DEFF: Design effect
DIGGI: Partially digital version of EMOTION intervention
EBI: Evidence-Based Intervention
EBP: Attitudes towards evidence-based practice
EBPAS: The Evidence-Based Practice Attitude Scale
EMOTION: Coping Kids Managing Anxiety and Depression Program
ERC: The Emotion Regulation Checklist
FAD: Family Assessment Device
GDPR: General Data Protection Regulation
GSE: General Self-Efficacy Scale
HSCL-10: Hopkins Symptoms Checklist
ICC: Intra-class correlation coefficient
ICS: Implementation Climate Scale
ILS: Implementation Leadership Scale
KINDL: Children quality of life questionnaire [Kinder Lebensqualität Fragebogen]
MASC-C/P: Multidimensional Anxiety Scale for children/parent version
MFS: Measurement Feedback System
PIE: Post-intervention questionnaire
PARCADS: Parenting to Reduce Child Anxiety and Depression Scale
PBI: Parental Bonding Instrument
RBUP: Regional center for child and adolescent mental health, east and south
RCADS: Revised Child Anxiety and Depression Scale
RCT: Randomized Controlled Trial
RKBU: Regional center for child and adolescent mental health and child welfare
SD: Standard Deviation
SMFQ: Mood and Feeling Questionnaire – Short form
TRF: Teacher’s Report Form
TWQ: Total Workload Questionnaire
VR: Virtual Reality

## References

[1] Merikangas KR, He J-P, Burstein M, Swanson SA, Avenevoli S, Cui L (2010). Lifetime prevalence of mental disorders in U.S. adolescents: Results from the National Comorbidity Survey Replication-Adolescent Supplement (NCS-A). J Am Acad Child Adolesc Psychiatry..

[2] Costello E, Mustillo S, Erkanli A, Keeler G, Angold A (2003). Prevalence and development of psychiatric disorders in childhood and adolescence. Arch Gen Psychiatry.

[3] Folkehelseinstituttet. Helsetilstanden i Norge. Folkehelserapporten – kortversjon. Oslo: Folkehelseinstituttet; 2018.

[4] Balazs J, Miklosi M, Kereszteny A, Hoven CW, Carli V, Wasserman C (2013). Adolescent subthreshold-depression and anxiety: Psychopathology, functional impairment and increased suicide risk. J Child Psychol Psychiatry.

[5] Nygaard E, Kårikstad V. Prioriteringsveileder psykisk helsevern for barn og unge Oslo: Helsedirektoratet; 2015.

[6] Martinsen K, Stark KD, Rodriguez K, Kendall PC, Arora P (2014). Mestrende barn: foreldremanual.

[7] Patras J, Martinsen KD, Holen S, Sund AM, Adolfsen F, Rasmussen LP (2016). Study protocol of an RCT of EMOTION: An indicated intervention for children with symptoms of anxiety and depression. BMC Psychol.

[8] Loevaas MES, Lydersen S, Sund AM, Neumer SP, Martinsen KD, Holen S (2020). A 12-month follow-up of a transdiagnostic indicated prevention of internalizing symptoms in school-aged children: the results from the EMOTION study. Child Adolesc Psychiatry Ment Health.

[9] Martinsen K, Rasmussen LMP, Wentzel-Larsen T, Holen S, Sund AM, Lovaas MES, et al. Prevention of anxiety and depression in school children: Effectiveness of the transdiagnostic EMOTION program. J Consult Clin Psychol. 2018. 10.1037/ccp0000360 30550301

[10] Rasmussen L-MP, Patras J, Neumer S-P, Adolfsen F, Martinsen KD, Holen S (2020). Facilitators and barriers to the implementation of EMOTION: an indicated intervention for young schoolchildren. Scand J Educ Res..

[11] Mychailyszyn MP, Brodman DM, Read KL, Kendall PC (2012). Cognitive-behavioral school-based interventions for anxious and depressed youth: a meta-analysis of outcomes. Clin Psychol.

[12] Tollefsen TK, Darrow SM, Lohne V, Berg-Nielsen TS (2020). Experiences with using an idiographic assessment procedure in primary mental health care services for adolescents. Int J Qual Stud Health Well-being.

[13] Bergman H, Kornør H, Nikolakopoulou A, Hanssen‐Bauer K, Soares‐Weiser K, Tollefsen TK, et al. Client feedback in psychological therapy for children and adolescents with mental health problems. Cochrane Database Syst Rev. 2018(8). 10.1002/14651858.CD011729.pub2 PMC6513116 30124233

[14] Lyon AR, Lewis CC, Boyd MR, Hendrix E, Liu F (2016). Capabilities and characteristics of digital measurement feedback systems: results from a comprehensive review. Adm Policy Ment Health.

[15] Hollis C, Falconer CJ, Martin JL, Whittington C, Stockton S, Glazebrook C (2017). Annual research review: digital health interventions for children and young people with mental health problems—a systematic and meta-review. J Child Psychol Psychiatry.

[16] Brendel KE, Maynard BR (2014). Child–parent interventions for childhood anxiety disorders: a systematic review and meta-analysis. Res Soc Work Pract.

[17] Silverman WK, Pina AA, Viswesvaran C (2008). Evidence-based psychosocial treatments for phobic and anxiety disorders in children and adolescents. J Clin Child Adolesc Psychol.

[18] Collins LM, Dziak JJ, Kugler KC, Trail JB (2014). Factorial experiments: efficient tools for evaluation of intervention components. Am J Prev Med.

[19] Freeman D, Reeve S, Robinson A, Ehlers A, Clark D, Spanlang B (2017). Virtual reality in the assessment, understanding, and treatment of mental health disorders. Psychol Med.

[20] Campbell MK, Elbourne DR, Altman DG (2004). CONSORT statement: extension to cluster randomised trials. BMJ.

[21] Angold A, Costello EJ, Messer SC, Pickles A (1995). Development of a short questionnaire for use in epidemiological studies of depression in children and adolescents. Int J Methods Psychiatr Res.

[22] Rhew IC, Simpson K, Tracy M, Lymp J, McCauley E, Tsuang D (2010). Criterion validity of the Short Mood and Feelings Questionnaire and one- and two-item depression screens in young adolescents. Child Adolesc Psychiatry Ment Health.

[23] March JS, Parker JDA, Sullivan K, Stallings P, Conners CK (1997). The Multidimensional Anxiety Scale for Children (MASC): factor structure, reliability, and validity. J Am Acad Child Adolesc Psychiatry.

[24] Olason DT, Sighvatsson MB, Smami J (2004). Psychometric properties of the Multidimensional Anxiety Scale for Children (MASC) among Icelandic schoolchildren. Scand J Psychol.

[25] Villabø M, Gere M, Torgersen S, March JS, Kendall PC (2012). Diagnostic efficiency of the child and parent versions of the Multidimensional Anxiety Scale for Children. J Clin Child Adolesc Psychol Off J Soc Clin Child Adolesc Psychol Am Psychol Assoc Div.

[26] Angold A, Erkanli A, Silberg J, Eaves L, Costello E (2002). Depression scale scores in 8–17-year-olds: effects of age and gender. J Child Psychol Psychiatry.

[27] Weisz JR, Vaughn-Coaxum RA, Evans SC, Thomassin K, Hersh J, Lee EH (2019). Efficient monitoring of treatment response during youth psychotherapy: the behavior and feelings survey. J Clin Child Adolesc Psychol Off J Soc Clin Child Adolesc Psychol Am Psychol Assoc Div.

[28] Martinsen K, Kendall PC, Stark K, Rodriguez K, Arora P (2017). Mestrende barn : gruppeledermanual barn.

[29] Martinsen K, Stark K, Rodriguez K, Kendall PC, Arora P (2017). Mestrende barn : gruppeledermanual foreldre.

[30] ECHO. Hvordan støtte barnet ditt? En veileder til foreldre med triste og engstelige barn. [Brochure]. Trondheim: RKBU, NTNU; 2019.

[31] Chorpita BF, Yim L, Moffitt C, Umemoto LA, Francis SE (2000). Assessment of symptoms of DSM-IV anxiety and depression in children: a revised child anxiety and depression scale. Behav Res Ther.

[32] Faul F, Erdfelder E, Buchner A, Lang AG (2009). Statistical power analyses using G*Power 3.1: tests for correlation and regression analyses. Behav Res Methods..

[33] Ukoumunne OC, Gulliford MC, Chinn S, Sterne JA, Burney PG. Methods for evaluating area-wide and organisation-based interventions in health and health care: a systematic review. Health Technol Assess. 1999;3(5). 10982317

[34] Holen S, Waaktaar T, Lervag A, Ystgaard M (2012). The effectiveness of a universal school-based programme on coping and mental health: a randomised, controlled study of Zippy's Friends. Educ Psychol.

[35] Altman DG (1991). Practical statistics for medical research.

[36] Beck AT, Brown GK, Steer RA. BDI-II, Beck depression inventory: manual. San Antonio, Tex.: Psychological Corp.; 1996. VI, 38 s. p.

[37] Runyon MK, Steer RA, Deblinger E (2009). Psychometric characteristics of the beck self-concept inventory for youth with adolescents who have experienced sexual abuse. J Psychopathol Behav Assess.

[38] Schwarzer R, Jerusalem M (2010). The general self-efficacy scale (GSE). Anxiety Stress Coping.

[39] Romppel M, Herrmann-Lingen C, Wachter R, Edelmann F, Düngen HD, Pieske B, et al. A short form of the General Self-Efficacy Scale (GSE-6): development, psychometric properties and validity in an intercultural non-clinical sample and a sample of patients at risk for heart failure. Psycho-Soc Med. 2013;10 10.3205/psm000091 PMC3578200 23429426

[40] Ravens-Sieberer U, Gortler E, Bullinger M (2000). Subjective health and health behavior of children and adolescents–a survey of Hamburg students within the scope of school medical examination. Gesundheitswesen.

[41] Haraldstad K, Richter J. Måleegenskaper ved den norske versjonen av KIDSCREEN. PsykTestBarn. 2014.

[42] Chorpita BF, Moffitt CE, Gray J (2005). Psychometric properties of the Revised Child Anxiety and Depression Scale in a clinical sample. Behav Res Ther.

[43] Rapee RM, Wignall A, Sheffield J, Kowalenko N, Davis A, McLoone J (2006). Adolescents' reactions to universal and indicated prevention programs for depression: perceived stigma and consumer satisfaction. Prev Sci.

[44] Tollefsen TK, Neumer S-P, Berg-Nielsen TS (2020). “What matters to you?”: a randomized controlled effectiveness trial, Using Systematic Idiographic Assessment as an intervention to Increase Adolescents’ perceived control of their mental health. J Adolesc.

[45] Achenbach TM, Rescorla L. Manual for the ASEBA school-age forms & profiles: child behavior checklist for ages 6–18, teacher's report form, youth self-report: an integrated system of multi-informant assessment. Burlington, Vt.: ASEBA; 2001. XII, 238 s. p.

[46] Backer-Grøndahl A, Martinussen M. Måleegenskaper ved den norske versjonen av Brief Problem Monitor (BPM). PsykTestBARN [Internet]. 2018; 8(1). Available from: https://psyktestbarn.r-bup.no/no/artikler/bpm-brief-problem-monitor.

[47] Martinussen M, Adolfsen F, Lauritzen C, Richardsen AM (2012). Improving interprofessional collaboration in a community setting: relationships with burnout, engagement and service quality. J Interprof Care.

[48] Mårdberg B, Lundberg U, Frankenhaeuser M (1990). The total workload of parents employed in white-collar jobs: construction of a questionnaire and scoring system.

[49] Ehrhart MG, Aarons GA, Farahnak LR (2014). Assessing the organizational context for EBP implementation: the development and validity testing of the Implementation Climate Scale (ICS). Implement Sci.

[50] Egeland KM, Skar A-MS, Endsjø M, Laukvik EH, Bækkelund H, Babaii A (2019). Testing the leadership and organizational change for implementation (LOCI) intervention in Norwegian mental health clinics: a stepped-wedge cluster randomized design study protocol. Implement Sci..

[51] Aarons GA, Ehrhart MG, Farahnak LR (2014). The implementation leadership scale (ILS): development of a brief measure of unit level implementation leadership. Implement Sci.

[52] Fixsen DL, Blase KA, Naoom SF, Wallace F (2009). Core implementation components. Res Soc Work Pract.

[53] Bird HR, Shaffer D, Fisher P, Gould MS (1993). The Columbia Impairment Scale (CIS): Pilot findings on a measure of global impairment for children and adolescents. Int J Methods Psychiatr Res.

[54] Bird HR, Andrews H, Schwab-Stone M, Goodman S, Dulcan M, Richters J (1996). Global measures of impairment for epidemiologic and clinical use with children and adolescents. Int J Methods Psychiatr Res.

[55] Sim WH, Jorm AF, Lawrence KA, Yap MBH (2019). Development and evaluation of the Parenting to Reduce Child Anxiety and Depression Scale (PaRCADS): assessment of parental concordance with guidelines for the prevention of child anxiety and depression. PeerJ..

[56] Epstein NB, Baldwin LM, Bishop DS (1983). The McMaster family assessment device. J Marital Fam Ther.

[57] Byles J, Byrne C, Boyle MH, Offord DR (1988). Ontario Child Health Study: reliability and validity of the general functioning subscale of the McMaster Family Assessment Device. Fam Process.

[58] Parker G, Tupling H, Brown L (2011). A parental bonding instrument. Br J Med Psychol.

[59] Cox BJ, Enns MW, Clara IP (2000). The Parental Bonding Instrument: confirmatory evidence for a three-factor model in a psychiatric clinical sample and in the National Comorbidity Survey. Soc Psychiatry Psychiatr Epidemiol.

[60] Rimehaug T, Wallander J, Berg-Nielsen T (2011). Group and individual stability of three parenting dimensions. Child Adolesc Psychiatry Ment Health.

[61] Kleppang AL, Hagquist C (2016). The psychometric properties of the Hopkins Symptom Checklist-10: a Rasch analysis based on adolescent data from Norway. Fam Pract.

