# Supplementary material for: Study protocol of a factorial trial ECHO: optimizing a group-based school intervention for children with emotional problems
Source: BMC Psychol. 2021 Jun 21;9:97. doi: 10.1186/s40359-021-00581-y (PMC8215478; doi:10.1186/s40359-021-00581-y)
Supplement: Supplementary file 1 — Additional file 1. Post-intervention questionnaire (PIE). [file 40359_2021_581_MOESM1_ESM.docx]

**Post Intervention Evaluation (PIE)**

**Participation, supervision, and general satisfaction**

| **Items** | **Response categories** | | | | | | | | |
| --- | --- | --- | --- | --- | --- | --- | --- | --- | --- |
| 1. Were you a group leader for EMOTION groups in the current semester? | Yes | | | No | |  | | | |
| 1. In which school was the group conducted? | *[List of participating schools]* | | | | | | | | |
| 1. The group I ran had: (Multiple choices allowed) | MFS | | | 16 sessions | | 8 + 8 sessions | | Parent meetings | |
| 1. Were you a group leader for more than one group the current semester? | Yes | | | No | |  | |  | |
| 1. In which other school was the group conducted? | *[List of participating schools]* | | | | | | | | |
| 1. Have you received supervision while running EMOTION-groups? | Yes | | | No | |  | | | |
| 1. Approximately how many hours of supervision have you received during the time you were a group leader? | 1 2 3 4 5 6 7 8 9 10 | | | | | | | | |
| 1. In general, how satisfied are you with the supervision you received from your supervisor in EMOTION? | Very  dis-satisfied | Dis- satisfied | | | Neutral | | Satisfied | | Very  satisfied |
| 1. In general, how satisfied are you with being a group leader in EMOTION? | Very  dis-satisfied | Dis- satisfied | | | Neutral | | Satisfied | | Very  satisfied |
| 1. In general, how satisfied are you with the manual in EMOTION? | Very  dis-satisfied | Dis- satisfied | | | Neutral | | Satisfied | | Very  satisfied |
| 1. Do you have other comments regarding the manual? | Yes | | No | | | | | | |
| Please specify: | *[Open text field]* | | | | | | | | |
| 1. If it was up to you, how likely is it that you will run EMOTION-groups in the future (after the project period)? | Very unlikely | Unlikely | | | Neither nor | | Likely | | Very likely |
| 1. Is there anything, e.g., heavy workload, which could hinder you from running groups next semester? | Yes | No | | |  | |  | |  |
| Please specify: | *[Open text field]* | | | | | | | | |

**Experiences with the different conditions in the ECHO-study:**

| **Use of Measurement Feedback System (MFS).**  Below are some statements regarding use of the feedback system MFS (MFS = the “MittEcho” app + publication portal). Please state your opinion by marking the alternative that fits you the best. | | | |
| --- | --- | --- | --- |
|  | | | |
| It was easy to use the feedback system (log in, retrieve data etc.). | Not at  all | 1 2 3 4 5 | To a very large extent |
| It was easy to apply for access to the children`s results on the publication portal mittecho.uio.no | Not at  all | 1 2 3 4 5 | To a very large extent |
| The feedback system (MFS) made it easier for me as a group leader to adapt the intervention to each child. | Not at  all | 1 2 3 4 5 | To a very large extent |
| The feedback system (MFS) was a useful tool to adjust the goals for each child. | Not at  all | 1 2 3 4 5 | To a very large extent |
| The feedback system (MFS) improves the help provided to the children. | Not at  all | 1 2 3 4 5 | To a very large extent |
| In general, I am satisfied with using the feedback system (MFS) in relation to the completion of the EMOTION program. | Not at  all | 1 2 3 4 5 | To a very large extent |
| Other comments: *[Open text field]* | | | |

| **Use of the digital version of EMOTION (DIGGI).**  Below are some statements regarding use of DIGGI, the digital version of the program. Please state your opinion by marking the alternative that fits you the best. | | | |
| --- | --- | --- | --- |
|  | | | |
| DIGGI was a useful resource. | Not at  all | 1 2 3 4 5 | To a very large extent |
| My impression is that the children completed DIGGI (between the regular sessions). | Not at  all | 1 2 3 4 5 | To a very large extent |
| My impression is that the children understood how DIGGI should be used. | Not at  all | 1 2 3 4 5 | To a very large extent |
| The children expressed that DIGGI was educational. | Not at  all | 1 2 3 4 5 | To a very large extent |
| It was easy to follow the children`s progression in DIGGI. | Not at  all | 1 2 3 4 5 | To a very large extent |
| Using DIGGI is a good approach to work with sad and anxious children. | Not at  all | 1 2 3 4 5 | To a very large extent |
| In general, I am satisfied with using DIGGI in relation to the completion of the EMOTION program. | Not at  all | 1 2 3 4 5 | To a very large extent |
| Other comments: *[Open text field]* | | | |

| **Use of the parent manual of EMOTION** | | | | | | | | |
| --- | --- | --- | --- | --- | --- | --- | --- | --- |
| In general, how satisfied are you with being a group leader in the parent meetings of EMOTION? | | Not at  all | | 1 2 3 4 5 | | | To a very large extent | |
| The parent meetings did the work with sad and anxious children better. | | Not at  all | | 1 2 3 4 5 | | | To a very large extent | |
|  | |  | |  | | |  | |
| In general, how satisfied are you with the parent manual in EMOTION? | | Very dissatisfied | Dis-satisfied | | Neutral | Satisfied | | Very satisfied |
| Do you have other comments to the parent manual? | *[Open text field]* | | | | | | | |

| **Item** | **Response categories** | |
| --- | --- | --- |
| Did you use VR-glasses in the group? | Yes | No |

| **Use of Virtual Reality (VR) during behavioral experiments.**  Below are some statements regarding use of virtual reality (VR) technology in the groups. Please state your opinion by marking the alternative that fits you the best. | | | |
| --- | --- | --- | --- |
|  | | | |
| The VR-glasses were a useful tool in the completion of the program. | Not at  all | 1 2 3 4 5 | To a very large extent |
| The VR-glasses made it easier for me as a group leader to adapt the intervention the children. | Not at  all | 1 2 3 4 5 | To a very large extent |
| It was easy to use the VR-glasses in the sessions. | Not at  all | 1 2 3 4 5 | To a very large extent |
| The children understood how it should be done. | Not at  all | 1 2 3 4 5 | To a very large extent |
| The children thought it was educational. | Not at  all | 1 2 3 4 5 | To a very large extent |
| The children thought it was fun. | Not at  all | 1 2 3 4 5 | To a very large extent |
| Using VR-glasses did my work with sad and anxious children better. | Not at  all | 1 2 3 4 5 | To a very large extent |
| In general, I am satisfied with using the VR-glasses in relation to the completion of the EMOTION program. | Not at  all | 1 2 3 4 5 | To a very large extent |
| Other comments: *[Open text field]* | | | |

# **Cost-benefit**

| **Items** | **Response categories** | | | |
| --- | --- | --- | --- | --- |
| How much time did you spend on: |  |  |  |  |
| 1. Preparations for one group session? | Less than 1 hour | 1 to 2 hours | 2 to 3 hours | More than 3 hours |
| 1. Supplementary work after one session? | Less than 1 hour | 1 to 2 hours | 2 to 3 hours | More than 3 hours |
| 1. Completion of one session? | Less than 1 hour | 1 to 2 hours | 2 to 3 hours | More than 3 hours |
