# Supplementary material for: Study protocol of a factorial trial ECHO: optimizing a group-based school intervention for children with emotional problems
Source: BMC Psychol. 2021 Jun 21;9:97. doi: 10.1186/s40359-021-00581-y (PMC8215478; doi:10.1186/s40359-021-00581-y)
Supplement: Supplementary file 3 — Additional file 3. COVID-19 questions parents. [file 40359_2021_581_MOESM3_ESM.docx]

**Questions related to the Corona pandemic - CHILDREN**

1) Have you been in contact with a doctor or school health nurse about the Coronavirus?

- Yes
- No

2) Have you, or anyone in your family, been infected with Corona?

- Yes
- No
- I don`t know

Indicate the degree to which the statements below are true for you:

|  | Not at all | To a small degree | To some degree | To a large degree | To a very large degree |
| --- | --- | --- | --- | --- | --- |
| 3) I have spoken a lot to my parents / guardians about Corona |  |  |  |  |  |
| 4) I have paid a lot of attention to news about Corona |  |  |  |  |  |
| 5) I have worried a lot about Corona |  |  |  |  |  |
| 6) Corona has made me more worried than I used to be |  |  |  |  |  |
| 7) Home-schooling has been a positive experience for me |  |  |  |  |  |
| 8) I felt lonely during the Corona pandemic |  |  |  |  |  |

9) Which strategy have you used when you felt worried about Corona?

- Thought of something fun to do or tried something new
- Done something calm and relaxing
- Talked to others
- Done an activity where I used? energy
- Tried to think less negative and more positive
- I haven’t been worried about Corona
- Other: (open text field)
